# Dynamic Profiling of Cell Free Tumour DNA in Aggressive B‐Cell Lymphoma From Diagnosis to Transformation at Relapse

**DOI:** 10.1002/jha2.70126

**Published:** 2025-08-19

**Authors:** Mark Waltham, Jonathan Wong, Galina Polekhina, Tessa Potezny, Pranav Dorwal, Daniella Brasacchio, Belinda Maher, Aditya Tedjaseputra, Gareth P. Gregory, Stephen Opat, Jake Shortt

**Affiliations:** ^1^ Department of Medicine School of Clinical Sciences at Monash Health Monash University Clayton Victoria Australia; ^2^ Monash Haematology Monash Health Clayton Victoria Australia; ^3^ Department of Epidemiology and Preventative Medicine School of Public Health and Preventative Medicine Monash University Melbourne Victoria Australia; ^4^ Monash Pathology Monash Health Clayton Victoria Australia; ^5^ Sir Peter MacCallum Department of Oncology University of Melbourne Melbourne Victoria Australia

**Keywords:** circulating tumour DNA, diffuse large B‐cell lymphoma, early molecular response, lymphoma diagnosis, NGS sequencing

## Abstract

**Background:**

Next generation sequencing (NGS) of cell free tumour DNA (ctDNA) provides a snapshot of lymphoma mutations correlating with tumour burden. We evaluated the ability of ctDNA to molecularly profile and track disease burden in patients with aggressive‐B‐cell lymphoma.

**Methods:**

Patients were prospectively recruited from the ‘standard‐of‐care’ clinic based on high‐risk clinical features. Using error‐corrected, capture‐based NGS of ctDNA, we profiled 17 patients receiving treatment for diffuse large B‐cell lymphoma to determine the utility of molecular profiling and dynamic response assessment through to measurable residual disease (MRD) states.

**Results:**

Somatic mutations were detected at baseline in 17/17 patients, including 13 with phased variants that could be leveraged for enhanced assay sensitivity. Early molecular response (EMR; ≥ 2 log_10_ reduction in ctDNA during cycle 1) was predictive of end‐of‐treatment complete metabolic response (CMR; EMR rate 100% vs. no EMR 12.5%, *p* = 0.0014) and 24‐month progression free survival (PFS; no progression events for those in EMR vs. no EMR 4.57 months, *p* = 0.015). Only 1/12 MRD‐negative patients relapsed. Interestingly, all three late relapses demonstrated histological infidelity and potential clonal evolution based on ctDNA results.

**Conclusion:**

These data further support the potential utility of molecular lymphoma profiling by ctDNA analysis and the predictive value of EMR as reported by independent groups. We also posit that ctDNA alone is insufficient to diagnose late relapses, where shared mutations may associate with differential histopathology.

## 1 Introduction

Diffuse large B‐cell lymphoma (DLBCL) and associated aggressive B‐cell lymphoma variants demonstrate marked genomic heterogeneity [1, 2, 3]. Tools like the revised International Prognostic Index (R‐IPI) prognosticate using readily available clinical parameters [4]. Histopathology also identifies poor‐risk features, including non‐germinal centre B‐cell (GCB), cell‐of‐origin (COO) and *cMYC* and/or *BCL2* expression [5]. Mutational correlates of COO performed on tissue provide more precise molecular subgrouping with prognostic implications [2]. However, routine tissue‐based genomic profiling is limited by access to sufficient diagnostic material and the applicability of serial invasive biopsies [6]. Moreover, biopsies from different anatomical sites may yield different molecular profiles in the context of spatial heterogeneity.

Small amounts of fragmented tumour DNA are present in the plasma of lymphoma patients (cell‐free tumour DNA; ctDNA), providing material for molecular analyses [6, 7]. The detection of immunoglobulin (IG) rearrangements provides a means to detect and monitor the majority of lymphomas bearing IG gene‐rearrangements but this methodology does not provide information on the somatic mutation profile [8]. In contrast, targeted hybridisation capture sequencing (e.g., the Cancer Personalised Profiling by deep‐Sequencing; CAPP‐Seq assay) allows mutation profiling and a quantitative lymphoma burden assessment [9, 10]. Notably, dynamic ctDNA responses are predictive of clinical responses. An ‘early molecular response’ (EMR; ≥ 2 log_10_ reduction by Day 21 of the first cycle) predicted 24‐month event‐free survival in patients receiving therapy for DLBCL [9, 11]. Moreover, the detection of measurable residual disease (MRD) by ctDNA may predict clinical relapse [10]. Thus, ctDNA may be deployed as a tool for risk‐adapted interventions to detect initial treatment failure or early relapse.

Here we prospectively applied an error‐corrected, hybrid capture‐based ctDNA assay to profile somatic mutations and copy number variations in a high‐grade B cell lymphoma cohort. Our results further support the predictive value of EMR and achieving MRD negativity. Of note, late relapses were characterised by mutational divergence and histological infidelity, including two cases of Hodgkin lymphoma (HL).

## 2 Methods

### 2.1 Study Design and Patients

Patients treated within the Monash Health network (Victoria, Australia) were prospectively enrolled. The study was approved by the Monash Health Human Research Ethics Committee (RES‐17‐0000050L). All patients provided written informed consent. Adults with DLBCL and high‐grade B‐cell lymphoma (HGBL) as defined by the 2016 revision of the WHO [12] with poor‐risk features (e.g., high R‐IPI [4], non‐GCB COO or relapsed disease) were included. Transformed low‐grade lymphoma was permitted. To reduce the potential for confounding by receipt of attenuated therapy, patients were required to have Eastern Cooperative Oncology Group (ECOG) 0–2 and be planned for treatment with curative intent.

### 2.2 Standard of Care Diagnostics and Response Assessments

Immunohistochemical (IHC) COO was determined by the Hans classifier [13]. *MYC* and *BCL2* protein expression were positive if ≥ 40% and ≥ 50% of tumour cells were stained by IHC respectively [5]. Fluorescence in situ hybridisation (FISH) for *MYC* and *BCL2* rearrangements were performed on selected cases (e.g., high Ki67 and *MYC* IHC positivity in GCB lymphomas). Investigator‐assessed responses were evaluated by fluorodeoxyglucose‐positron emission tomography (FDG‐PET), designated according to Lugano criteria [14].

### 2.3 Statistical Considerations

The target cohort size of 20 was pragmatically determined based on budget and projected accrual within 3 years. This would provide 87% power to detect a relapse rate of 45%, which we deemed sufficient for a pilot study where progression events would inform potential clinical utility. The pre‐specified primary endpoint was analytical: to develop plasma‐based assay for mutational profiling and MRD monitoring in the majority of DLBCL cases, with sensitivity to detect a variant allele frequency (VAF) of less than 0.5%. Survival endpoints were considered exploratory given the small sample size, with the 24‐month progression free survival (PFS) endpoint selected post hoc to align with the initial publication defining the predictive nature of attaining EMR at this milestone [9]. Overall survival (OS) and PFS were measured from initiation of therapy and calculated by Kaplan–Meier using Log‐rank analysis and Mantel–Haenszel for hazard ratio (HR) in GraphPad Prism (Ver 10). Relapse, progression or death from any cause were considered progression events. Median potential follow‐up was estimated by reversing the censor in Kaplan–Meier analysis of OS [15]. Linear relationships were quantified using Pearson's co‐efficient (*r*).

### 2.4 Sample Collection, NGS Library Synthesis and Sequencing

A schema indicating scheduling of blood draws is provided in Figure S1. In addition to baseline sampling and time‐points correlating end of treatment (EOT) PET assessments, an ‘early’ sample was planned during the first cycle and ‘late’ sample collected in remission after completion of therapy. Details for blood/buccal collection, processing and NGS library synthesis are provided in Supporting Information. Critically, minimal PCR cycles were used at both pre‐ and post‐hybridisation steps to maximise diversity of represented source DNA molecules, and unique molecular identifiers (UMI; 10 nt bp) were incorporated to enable digital error correction as we previously described [16, 17]. Forty‐two genes were selected for analysis based on frequency of mutation in DLBCL, differential mutation according to COO and targets of somatic hypermutation and chromosomal translocation (e.g., *MYC* and *BCL2*) [18] (Table S1).

### 2.5 NGS Data Analysis

The bioinformatic pipeline was as described previously [16], incorporating tumour/normal (buccal gDNA) and including UMI de‐duplexing and consensus calling using fgbio (Fulcrum Genomics /fgbio), bwa for alignment to human genome (hg19), VarDict [19] for variant calling and CNVkit for copy number analysis [20]. Variant calls were curated by inspecting BAM files in Integrative Genomics Viewer (IGV) [21]. Phased somatic variants (paired within 100 nucleotide pairs) were identified from BAM files and verified in IGV. NGS data is available in the NCBI/SRA repository under accession number PRJNA1197147.

## 3 Results

### 3.1 Baseline Genomic Profiling

**FIGURE 1:**
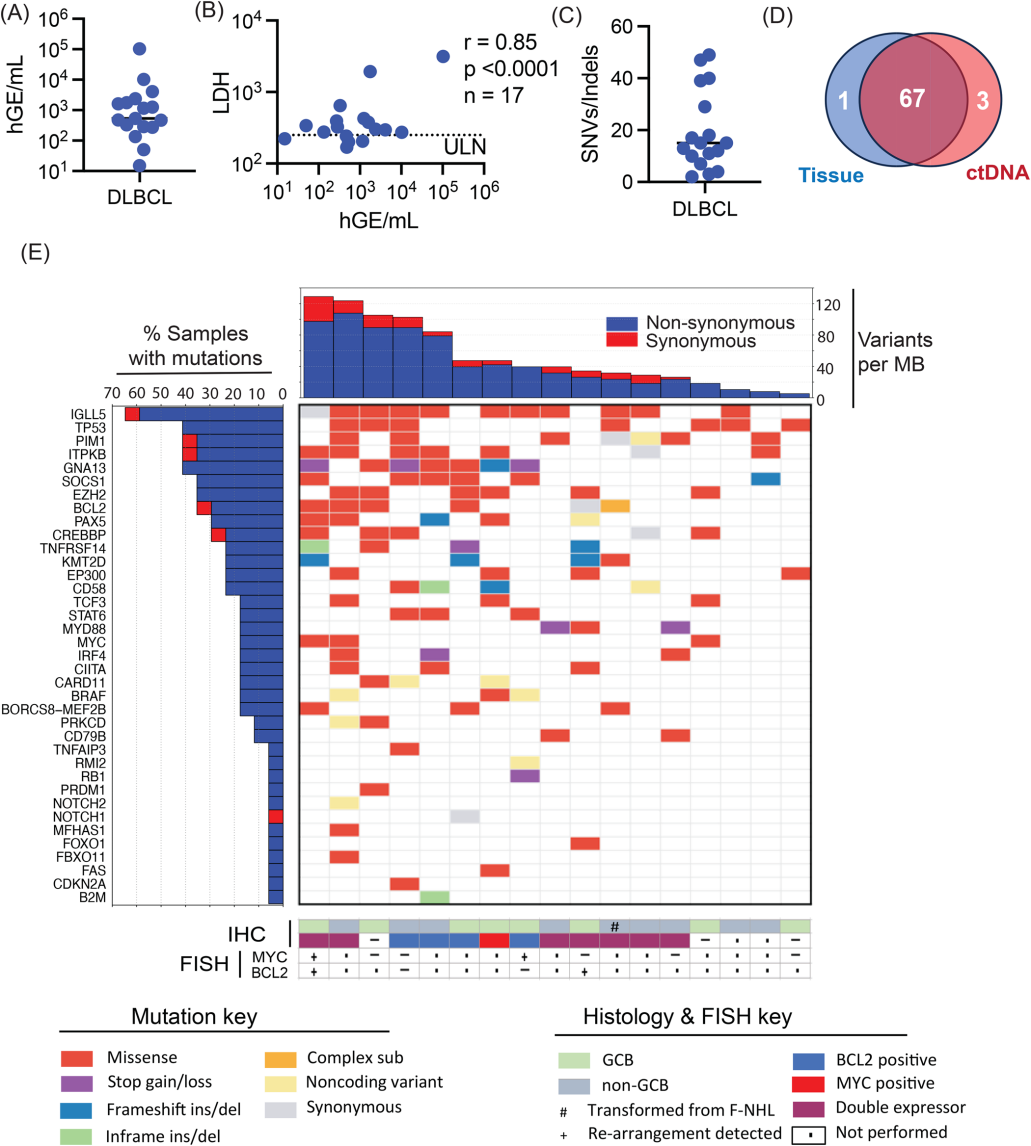
Baseline mutational profiling by ctDNA analysis. (A) ctDNA burden in the patients with DLBCL. (B) Correlation of ctDNA versus plasma LDH from patients in (A). (C) Frequency of mutations in baseline plasma samples. (D) Venn diagram comparing mutation detection in tumour biopsy compared to ctDNA for the patients with tumour sequencing. (E) Co‐mutation plot illustrating baseline variants for all patients evaluated in the study. DLBCL, Diffuse Large B‐cell lymphoma; F‐NHL, follicular non‐Hodgkin lymphoma; GCB, germinal centre B‐cell; hGE/mL, human genome equivalent per millilitre; LDH, lactate dehydrogenase; MB, megabase; ULN, upper limit of normal.

Characteristics of the 20 patients recruited to the study are presented in Table 1. Three patients were consented and underwent sample collection but did not meet inclusion criteria on clinicopathological review (one with primary CNS lymphoma without systemic involvement, and two with non‐transformed low‐grade disease) and were excluded from further analyses (Figure S2). Of the 17 remaining patients, 15 were recruited at the initiation of first‐line therapy and one at commencement of salvage; one patient who underwent surgical resection of initial disease sites was recruited after three cycles of R‐CHOP with disease recurrence suspected on interim FDG‐PET scanning. At the data censor date, median potential follow‐up was 50 months (range 1.6–61 months). Consistent with previous reports, pre‐treatment cell free DNA concentrations in patients with DLBCL were elevated (median 23.1 ng/mL; range 5–892 ng/mL, *n* = 17) relative to expected values for healthy controls (∼5 ng/mL) [6]. Following NGS sequencing, calculated DLBCL patient ctDNA values (median: 531 hGE/mL, IQR: 280–2072 hGE/mL) correlated with LDH levels (Figure 1A,B). However, neither the baseline ctDNA concentration threshold of 2.5 log_10_ hGE/mL [9], nor the R‐IPI [4] predicted PFS in the DLBCL patients (Figure S3). Spiking experiments demonstrated correct identification of 97/101 SNPs covered within the panel at a 0.2% dilution yielding an average sensitivity for VAF detection at 0.21% (Figure S4). Somatic mutations were detected in the baseline plasma of all DLBCL cases (median: 15, range: 2–49 mutations; Figure 1C–E). Phased variants (i.e., those detected in *cis* on the same cfDNA molecule) [18] were present in 13 patients (range 1–27 phased pairs; not shown). Whole exome sequencing on paired tumour samples was available in five DLBCL where there was 96% concordance between ctDNA and tissue for genes represented in the panel (Figure 1D and Figure S5). The ctDNA mutation profile of the cohort and results of standard of care diagnostics are provided in Figure 1E. The frequency of somatic variants detected was consistent with prior DLBCL sequencing efforts [1] and included a high rate in targets of aberrant somatic hypermutation [22] (e.g., *IGLL5*, *PIM1*), mutations in genes that convey therapeutic resistance (e.g., *TP53*) [23] and those associated with susceptibility to targeted therapies (e.g., *EZH2^Y646^*) [24].

**TABLE 1:** Patient demographics and clinical characteristics.

Characteristic	Patients (*n* = 17)
**Age, median (range)**	53 (18–79)
**Sex, *n* (%)**	
Female	6 (35)
**ECOG performance status, *n* (%)**	
0	7 (41)
1	10 (59)
2–4	0
**Extent of disease at study entry, *n* (%)**	
Stage I‐II	2 (12)
Stage III‐IV	15 (88)
B‐symptoms	7 (41)
Extranodal disease	12 (71)
Elevated LDH	10 (59)
**Revised International Prognostic Index, *n* (%)**	
Very good (0)	0
Good (1–2)	6 (35)
Poor (3–5)	11 (65)
**WHO subtype, *n* (%)**	
DLBCL	16 (94)
GCB	7 (41)
Non‐GCB	9 (53)
HGBL with MYC & BCL2 translocation	1 (6)
**Prior lines of systemic therapy, *n* (%)**	
0^a^	16 (94)
1	0
2	1 (6)
**Primary therapy administered, *n* (%)**	
R‐CHOP like	16 (94)
R‐CHOP (± MTX) (± RT) (± RM)	12 (71)
R‐CHOP then R‐DA‐EPOCH	1 (6)
R‐CHOP ± Polatuzumab/placebo^b^	3 (18)
Dinaciclib + pembrolizumab^b^	1 (6)

Abbreviations: DA‐EPOCH, dose adjusted etoposide, prednisolone, vincristine, cyclophosphamide, doxorubicin; DLBCL, diffuse large B‐cell lymphoma; ECOG, Eastern Cooperative Oncology Group; GCB, germinal centre B‐cell; HGBL, high grade B‐cell lymphoma; LDH, lactate dehydrogenase; MTX, high dose methotrexate; R‐CHOP, rituximab, cyclophosphamide, doxorubicin, vincristine, prednisolone; RM, rituximab maintenance; RT, consolidative radiotherapy.

^a^ Includes one patient recruited during first line R‐CHOP therapy.

^b^ Treated on clinical trial protocols.

**FIGURE 2:**
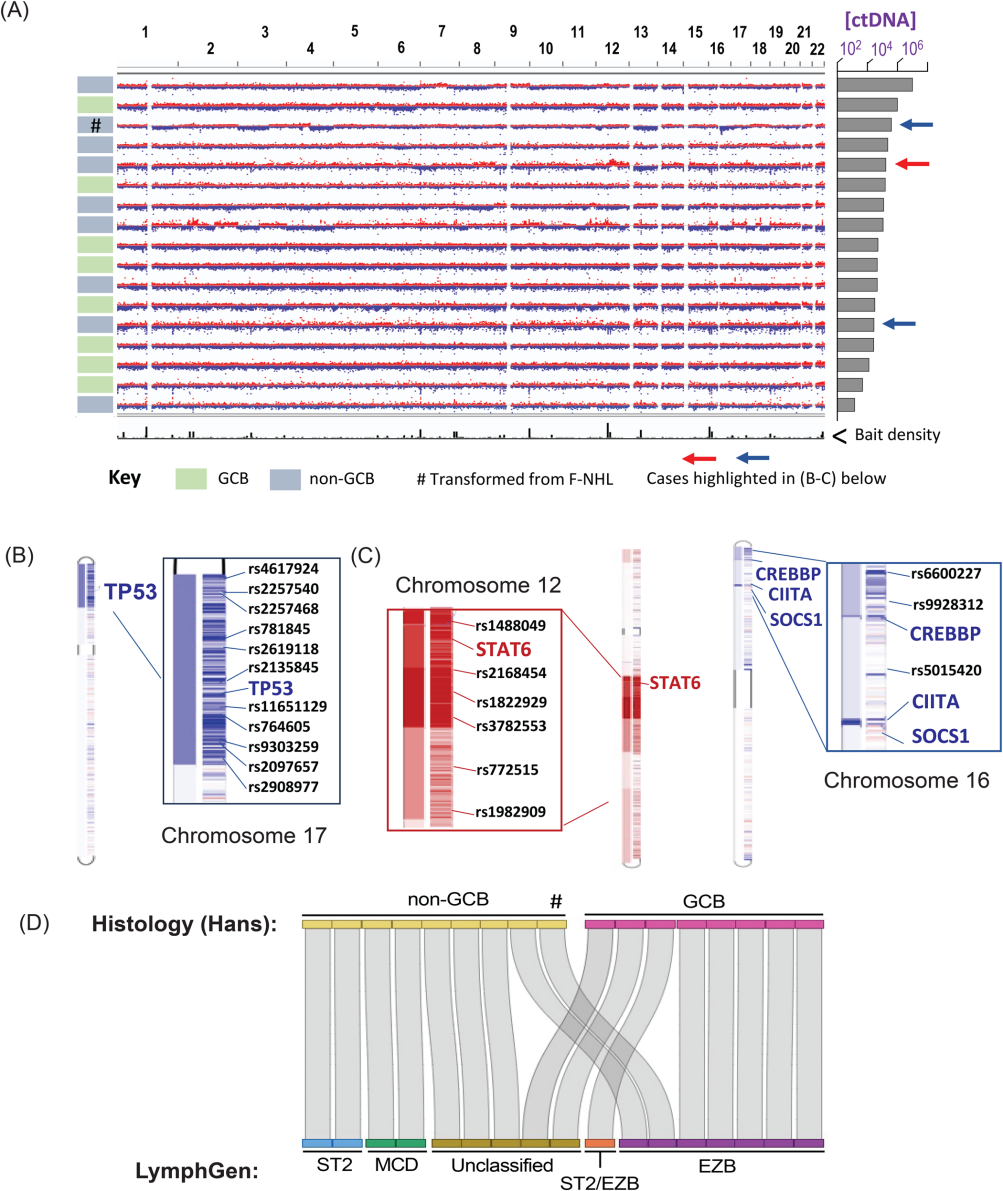
Copy number variations demonstrated in ctDNA plasma. (A) Copy number plot for patient samples by ctDNA concentration. Chromosome locations are annotated at the top of the plot. (B) Example of 17p copy number loss involving *TP53* locus corresponding to patient with transformed F‐NHL (third row from top in [A]). (C) Examples of recurrent copy number gains (chromosome 12q) and losses (chromosome 16p) annotated by rsID numbers for SNPs and genes of interest with highlighted by arrowed rows in (A). (D) Comparison of histological diagnosis to LymphGen classifier applied to ctDNA mutation profile.

Copy number variations were evident in the ctDNA from most patients (Figure 2A) including examples of 17p deletion (leading to loss‐of‐heterozygosity for mutant *TP53*; Figure 2B), and abnormalities segregating with COO (e.g., *CREBBP, CIITA* and *SOCS1* loss and *STAT6* amplification [25]; Figure 2C). We next combined the plasma ctDNA mutational and CNV profile to apply a molecular DLBCL classifier [2] (Figure 2D). A ‘LymphGen’ classification could be allocated for 12/17 DLBCLs and expectedly Hans‐designated GCB cases correlated well with EZB and ST2 subtypes (6/8 cases). The case of DLBCL that had transformed from F‐NHL classified as EZB, despite appearing as non‐GCB derived by Hans. Although MCD (*n* = 2) cases were exclusively in the non‐GCB group, the remainder of Hans non‐GCB cases were either unclassifiable or re‐allocated to GCB‐associated entities (ST2 and EZB).

### 3.2 Dynamic ctDNA Assessment and Outcomes

**FIGURE 3:**
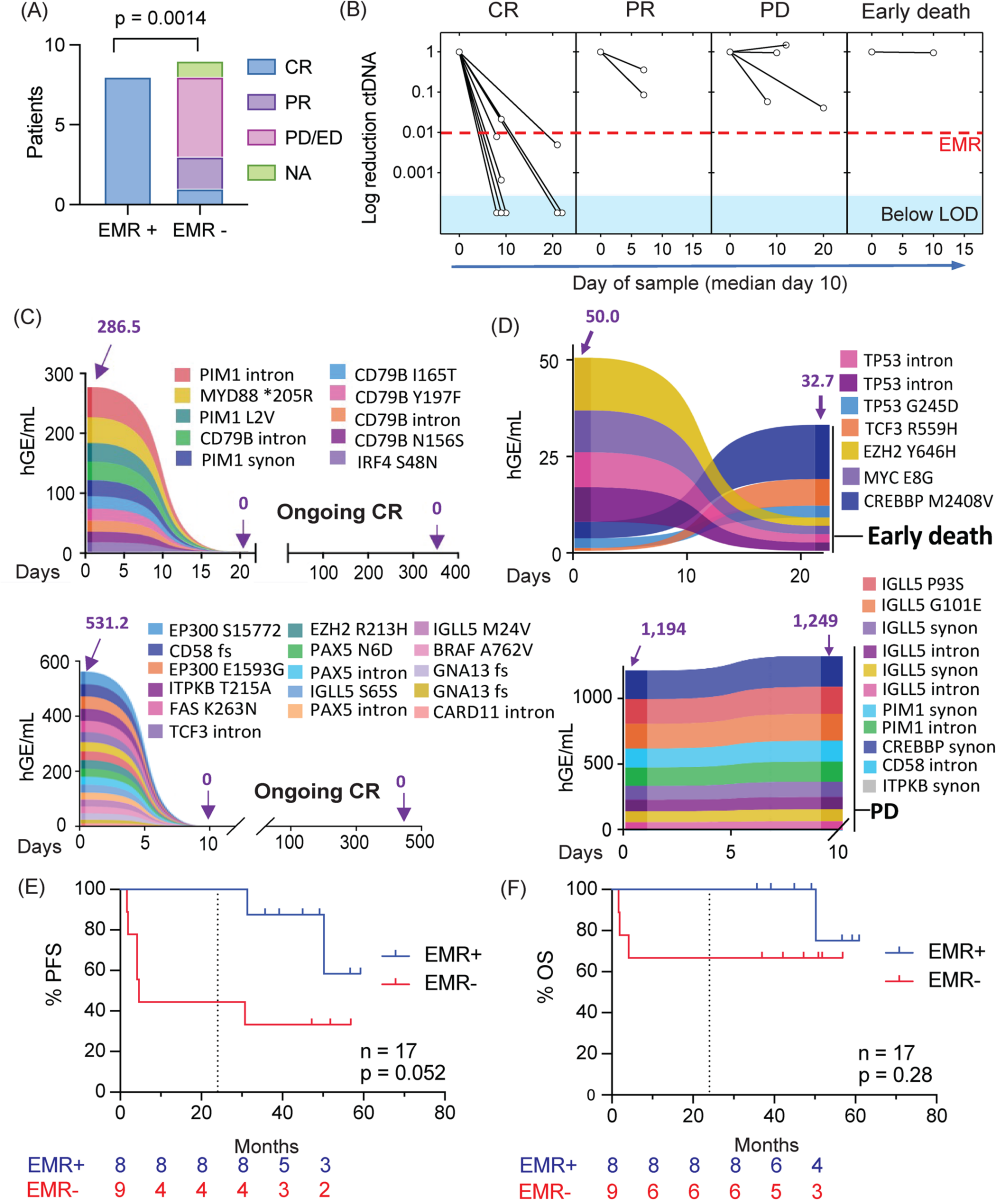
Early molecular response (EMR) in treatment outcomes. (A) End of treatment response stratified by EMR. (B) ctDNA changes from baseline according to clinical response. (C) Ribbon plot examples of two cases achieving EMR. (D) Ribbon plot examples of two cases without EMR. (E) Progression free survival (PFS) and (F) Overall survival (OS) for DLBCL patients stratified by EMR. Number of patients at risk are tabulated below each graph. CR, complete response; NA, not available; PD/ED, progressive disease/early death; PR, partial response.

To explore the predictive value of early ctDNA changes, we correlated the log‐fold change in ctDNA with EOT response assessments and PFS for the DLBCL patients. ‘Early’ ctDNA samples were collected at a median of 10 days (range: 7–22 days) post therapy. EMR was defined as a ≥ 2 log_10_ reduction in ctDNA at this time point based on a prior publication using CAPP‐Seq technology on Day 21 [9]. Of the 17 patients, all eight with EMR achieved complete metabolic response (CMR) on EOT staging. For the nine patients without EMR, one attained CMR at EOT with the remainder demonstrating partial metabolic responses (PR; *n* = 2), early death prior to staging (ED; *n* = 1) and/or early disease progression (PD) (*n* = 4) (Figure 3A,B). One patient from this group was in CMR on interim FDG‐PET but did not undergo EOT scanning (designated as not available; N/A in Figure 3A). Representative cases for patients with EMR versus ED/PD are provided in Figure 3C,D. Together these data indicate that attaining EMR was associated with EOT CMR.

Despite the failure of R‐IPI and baseline ctDNA concentration to predict PFS (Figure S3), EMR status predicted the 24‐month PFS threshold previously linked to ctDNA analysis [9]. At 24 months, none of the DLBCL patients who achieved EMR had relapsed whereas 56% of those without EMR had progressed (*p* = 0.015 Log‐rank; HR 0.109 [95% CI: 0.018–0.65]). This observation also held when analysis was restricted to the treatment‐naive patients recruited prior to initiation of initial therapy (*n* = 15; *p* = 0.0442 Log‐rank; HR 0.09 [95% CI: 0.0086 – 0.94]). With additional follow up to the censor date the median PFS was not reached for those with EMR versus 4.57 months for EMR failure (*p* = 0.0522 Log‐rank; HR 0.24 [95% CI: 0.057 – 1.014) (Figure 3E). Differences in OS stratified by EMR were non‐statistically significant (median OS not reached in either group; *p* = 0.28). Of note, three late (> 24 month) progression events were observed. However, all manifested with differential pathology including one case of F‐NHL, grade 3A and two cases of HL.

### 3.3 Molecular Persistence and Histological Transformation at Relapse

**FIGURE 4:**
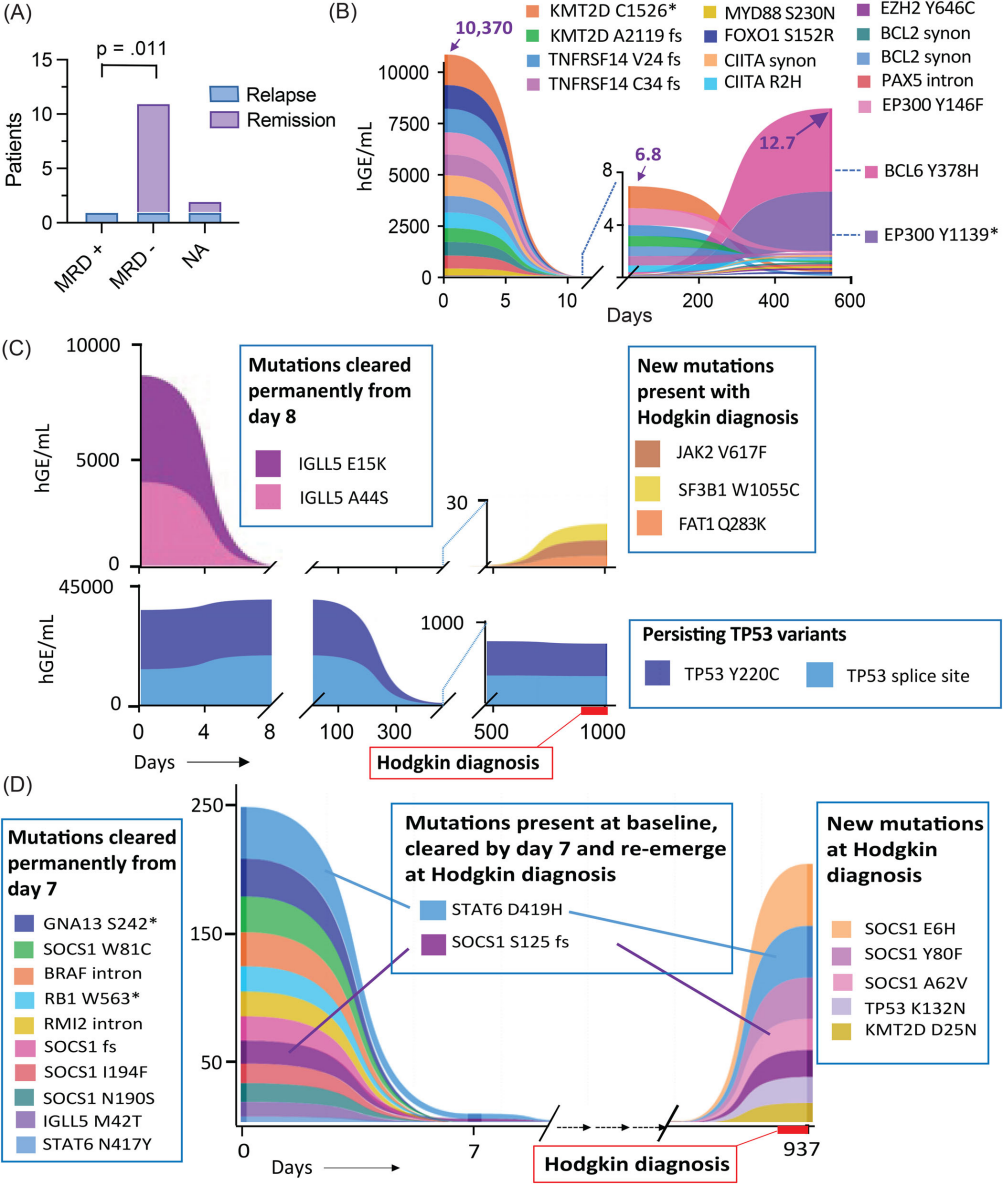
Measurable residual disease (MRD) and mutational profile of late relapses. (A) Relapse status stratified by detectable MRD in 12 DLBCL patients with samples collected in remission. (B) Ribbon plot of clonal dynamics in DLBCL patient who attained EMR but had detectable MRD prior to late F‐NHL relapse. (C) Ribbon plot of clonal dynamics for DLBCL patient with concurrent smoldering myeloma who attained EMR and MRD negativity with later relapse with Hodgkin lymphoma on a background of high‐risk (*TP53* mutated) clonal haematopoiesis. (D) Ribbon plot illustrating clonal dynamics for DLBCL patient who achieved CR on interim PET but did not undergo MRD testing and later relapsed with Hodgkin lymphoma.

We next evaluated the implications of positive MRD in patients successfully attaining CMR. Post‐remission MRD monitoring was available on 12 patients collected after a median 8.8 months (range 1.5–18 months) post confirmation of remission after initial therapy or salvage (Figure 4A; Note: Two patients were lost to follow‐up and others deceased early). One patient had evidence of MRD and experienced relapse with F‐NHL, 26 months following MRD detection (Figure 4B). All but one of the 11 MRD‐negative patients remained in remission at follow‐up. One HL relapse occurred 17 months following the negative MRD result (Figure 4C). Post remission MRD monitoring was unavailable on two patients in CR, one of whom also relapsed with HL (Figure 4D). Therefore, MRD negativity correlated with relapse free survival (Figure 4A).

Large B‐cell transformation of low‐grade lymphomas is well recognised with distinct models of clonal evolution described [26]. Late F‐NHL relapse following DLBCL treatment may indicate that the initial presentation represented transformed disease with subsequent relapse of the underlying low‐grade component [3]. Consistent with this hypothesis, the patient manifesting with late F‐NHL relapse originated from DLBCL with typical molecular and IHC profile of a GCB neoplasm with *BCL2* but not *MYC* re‐arrangements. Despite attaining both EMR and CMR with rapid elimination of multiple SNVs, this patient demonstrated new low level (12.7 hGE/mL) *BCL6* and *EP300* variants in ctDNA detected after 15 months of first CMR and 2 years prior to relapse (Figure 4B).

Our cohort was remarkable for histological variation with morphological features of classical HL in two late relapses after successful treatment for DLBCL. Clinicopathological features of these patients are provided in Table 2.

**TABLE 2:** Clinicopathological characteristics of patients with late Hodgkin relapse.

	Patient #02 (as depicted in Figure 4C)	Patient #16 (as depicted in Figure 4D)
Age/sex	69/M	24/F
Histology	DLBCL, non‐GCB	DLBCL, GCB
Cytogenetics/FISH	Not performed	MYC‐rearranged BCL2/BCL6 negative
Flow cytometry	Not performed	CD19^+^, CD20^+^, kappa light chain restricted
Ann Arbor stage	IVA	IVA
R‐IPI	Good	Poor
Mediastinal disease	No	Yes
EMR	Yes	No
Treatment	R‐CHOP	R‐CHOP, HD‐MTX, RT
Response to initial therapy	CMR	CMR^*^
MRD	Negative	NA
Concurrent diagnosis	Smoldering multiple myeloma	No

Abbreviations: CD, cluster of differentiation; CMR, complete metabolic response; DLBCL, diffuse large B‐cell lymphoma; EMR, early molecular response; FISH, fluorescence in situ hybridisation; GCB, germinal centre B cell; HD‐MTX, high dose methotrexate; MRD, measurable residual disease; NA, not available; R‐CHOP, rituximab, doxorubicin, vincristine, prednisolone; R‐IPI, revised international prognostic index; RT, radiotherapy.

* on interim FDG‐PET; did not undergo end‐of‐treatment PET.

The first was a 69‐year‐old male with smoldering myeloma diagnosed concurrent to non‐GCB DLBCL. Baseline exome sequencing of tumour demonstrated two *TP53* and two *IGLL5* variants that were concordant in plasma ctDNA and not present in germline DNA. Bone marrow examination demonstrated 30% plasma cells without evidence of DLBCL involvement (not shown); myeloma FISH studies were not performed. Treatment with R‐CHOP resulted in CMR. Of note, the *TP53* variants maintained a high VAF (∼40%) in plasma during and after treatment despite early clearance (EMR) of the *IGLL5* variants and sustained remission from DLBCL (Figure 4C). The patient subsequently relapsed with advanced stage classical HL, 44 months after initial therapy and was treated to CMR with adriamycin, vinblastine and dacarbazine followed by pembrolizumab. ctDNA analysis utilising an extended gene panel at Hodgkin relapse demonstrated persisting, high‐VAF *TP53* variants with new mutations in *SF3B1*, *JAK2* and *FAT1* but not the *IGLL5* mutations previously associated with DLBCL. The patient later developed therapy‐related acute myeloid leukaemia (AML) bearing the same *TP53* variants that had persisted unchanged in the plasma since his initial lymphoma presentation.

The second case was a 24‐year‐old female with *cMYC‐*rearranged GCB DLBCL, diagnosed on paraspinal mass biopsy (Figure S5). A concurrent mediastinal mass was not biopsied. She attained CMR on interim PET and completed R‐CHOP, consolidated with high‐dose methotrexate and mediastinal radiotherapy. No sample was collected for ctDNA analysis at completion of initial therapy, and she relapsed with classical HL after 2 years of remission. As shown in Figure 4D, 10 unique variants present at baseline were permanently cleared from the plasma from the time of EMR assessment. At the time of HL relapse, baseline *STAT6* and *SOCS1* variants re‐emerged along with three new variants in *SOCS1* and new *TP53* and *KMT2D* mutations.

## 4 Discussion

Here we show that capture‐based NGS of ctDNA on a series of DLBCL patients has utility in molecular and copy number profiling in most cases. ctDNA profiling complemented IHC in the inference of COO and identified mutations associated with therapeutic resistance (e.g., *TP53*) and potential applicability of novel therapies (e.g., *EZH2* hotspots). In the subset of patients with concurrent molecular profiling of tumour, there was high mutational concordance. These data are consistent with prior descriptions of the utility of liquid biopsy in the initial diagnostic work‐up of DLBCL [7, 9, 27].

Although limited by small numbers, our study was performed prospectively on a cohort with extended follow‐up, providing an opportunity to further evaluate the importance of dynamic changes in ctDNA as an early prognosticator. Samples for EMR assessment were collected a median of 10 days following treatment initiation. Limited statistical power was evidenced by the failure of baseline ctDNA burden and R‐IPI to predict PFS. Despite this, and consistent with previous reports [9, 11], EMR attainment was predictive of both EOT response and 24‐month PFS. Thus, our data further supports the original description of EMR [9] and its subsequent validation in a trial cohort of DLBCL patients [11]. The capacity to predict treatment failure within 21 days suggests risk‐adapted therapeutic changes to mitigate poor outcomes should be feasible by the second or third treatment cycle. A similar strategy incorporating chemointensification based on early PET responses failed to show benefit [28]. However, synthesis of the quantitative ctDNA response with a ctDNA mutational profile could inform treatment with a molecularly targeted adjunct to chemotherapy as is being evaluated in clinical trials [29]. Conversely, EMR could guide the safe de‐escalation of therapy for patients otherwise considered too high risk for this approach based on clinical parameters or equivocal PET responses [30]. In either case, integration of EMR to PET imaging is likely to further refine prognosis and optimise early risk‐adapted approaches.

The persistence or recrudescence of molecular MRD in clinical remission is another logical point of intervention to prevent relapse. PhasED‐seq has extremely high sensitivity (to 0.0001%), due to purposeful design capturing mutated genomic regions enriched for phased variants [18]. Our assay was designed prior to the description of PhasED‐Seq with genes selected based on frequency of mutation and relevance to biology of DLBCL but included a number of somatic hypermutation targets. Although we were able to detect phased variants in 13 out of 17 patients, the assay design was not bespoke to this purpose and so the remaining patients may have had phased variants that were not detected in our assay.

Postulated MRD eradication strategies include CD19‐targeted immune effector engaging therapies as have been successfully purposed in B‐cell acute lymphoblastic leukaemia [31]. However, certain aspects of ctDNA based MRD interpretation may confound this approach. Firstly, persisting variants that may represent ancestral clonal haematopoiesis of indeterminate potential (CHIP) and are therefore represented in both lymphoma and the haematopoietic stem cell compartment (HSC), require consideration. While this is likely to be more of an issue for peripheral T‐cell lymphomas due to the higher frequency of such mutations [16], variants in *TET2, DNMT3A* and *TP53* are also recurrent in DLBCL. In our series the case with non‐germline, persistent high VAF *TP53* variants in ctDNA concordant in both initial DLBCL tissue and later HL, *TP53* could not be used for the purposes of EMR or MRD monitoring. Definitive confirmation of ancestry within the HSC pool was an unfortunate consequence of later development of therapy‐related AML with the same *TP53* perturbations. Moreover, this HL ‘transformation’ had a distinct somatic mutation profile from the antecedent DLBCL indicating that the HL may have been a second primary malignancy arising out of the same high‐risk CHIP milieu, rather than a true histological transformation from parental DLBCL.

Composite lymphomas demonstrate features of both DLBCL and HL and have overlapping molecular features indicating a shared COO [32]. Hodgkin variant transformation of chronic lymphocytic leukaemia is a rare but well described phenomenon and patients may relapse with DLBCL following treatment for HL [33]. The second HL ‘transformation’ case showed recurrence of a subset of original mutations, along with new somatic variants (*SOCS1* and *KMT2D*) suggesting true clonal evolution. As this patient's initial DLBCL diagnosis was from paraspinal tissue, it is plausible in retrospect that the concurrent mediastinal disease had Hodgkin elements at baseline. Both cases highlight the importance of obtaining a definitive tissue biopsy at progression, irrespective of the re‐emergence of baseline ctDNA variants.

In conclusion, this exploratory series further supports the tractability of ctDNA‐based mutation profiling and the potential to predict treatment failure within the first cycle of chemoimmunotherapy for DLBCL. Prospective clinical trials should leverage EMR as a tool for risk‐adapted early intervention strategies.

## Author Contributions

J.S., G.P.G., S.O. and M.W. designed the study. J.W., T.P. and A.T. collected data. D.B. and B.M. performed sample preparation. M.W. performed ctDNA analyses. P.D. performed histological review. G.P. assisted with bioinformatic analyses and figure generation. All authors critically reviewed the manuscript and data.

## Conflicts of Interest

Jake Shortt reports research funding from Astex/Taiho, advisory board membership for Otsuka, Bristol Myers Squibb, Astellas, Novartis and speaker's fees from Mundipharma and Novartis. GPG reports research funding from BeiGene, Janssen and Merck and advisory board membership for Roche, Merck, Bristol Myers Squibb, Gilead Kite, Amgen, Clinigen and Prelude Therapeutics. Pranav Dorwal reports advisory board membership for Roche and Taiho. Stephen Opat's institution has received research funding from AbbVie, AstraZeneca, BeiGene, Gilead, Janssen, Pharmacyclics, Roche and Takeda. Stephen Opat has acted on advisory boards and received honoraria from AbbVie, Astra Zeneca, BeiGene, Gilead, Janssen and Merck. No authors have conflicts of interest directly related to the published work.

## Supporting information




**Figure S1**: Intended schedule for blood draws for plasma ctDNA analysis. **Figure S2**: Consort diagram of patients participating in the study. EOT, end of treatment. *includes patient who had baseline and EMR samples collected, missed EOT collection but continued to provide study samples at representation with Hodgkin relapse. **Figure S3**: (A) Progression free survival for all DLBCL/HGBL patients stratified by baseline ctDNA concentration. (B) Progression free survival for treatment naive DLBCL/HGBL patients stratified by R‐IPI (no patients had ‘very good’ R‐IPI designation). **Figure S4**: (A) Sensitivity and detection limit for collapsed UMI‐family data (sized 3 or more family members) for 0.2% mix of control DNA from two individuals (101 SNP sites represented). (B) To assess background error rates, nucleotide sites 3’ and 5’ of the interrogated SNPs were analysed for the most frequent ‘alt base’ call (202 sites). In only one instance was an alternate base called, representing an allele frequency approximately 8‐fold lower than the deemed limit of detection (0.2%). **Figure S5**: Comparison of variants detected by whole exome sequencing (WES) of tissue biopsy versus deep sequencing of ctDNA illustrated by individual subjects. Light blue, variants detected on the 42‐gene panel with ctDNA input; Light green, variants detected by WES of tissue biopsy for the same 42 genes; Dark green, additional variants detected by WES of tissue biopsy but not represented in the 42‐gene ctDNA panel. Also represented are the LymphGen classifications based on ctDNA and WES data. GCB versus non‐GCB status is annotated according to Hans classifier. **Figure S6**:Histological features of patient #16 at diagnosis with DLBCL and relapse with Hodgkin lymphoma (x400).

**Table S1**: Gene list (n=42) panel selected for assessment by ctDNA assay. An additional set of 1040 SNP genomic loci (distributed throughout the genome) was incorporated to facilitate CNV analysis. The inclusion of a SNP‐based CNV backbone also permitted the detection of mis‐labelled specimens using genotype information inherent to the sequence data to match samples as a quality control step. The total targeted region size for the design was 148.2 kbp, tiling density was ∼3x and total number of unique probes was 12,701.

eJHaem_Waltham et al_Supplementary Methods_R1.docx

## Data Availability

For sequencing data refer under Methods: *Bioinformatics and NGS data analysis*. The data that support the findings of this study are openly available in NCBI/SRA repository at https://www.ncbi.nlm.nih.gov/sra, reference number PRJNA1197147.
