# Supplementary material for: Dynamic Profiling of Cell Free Tumour DNA in Aggressive B‐Cell Lymphoma From Diagnosis to Transformation at Relapse
Source: EJHaem. 2025 Aug 19;6(4):e70126. doi: 10.1002/jha2.70126 (PMC12363405; doi:10.1002/jha2.70126)
Supplement: Supplementary file 1 — Figure S1: Intended schedule for blood draws for plasma ctDNA analysis. Figure S2: Consort diagram of patients participating in the study. EOT, end of treatment. *includes patient who had baseline and EMR samples collected, missed EOT collection but continued to provide study samples at representation with Hodgkin relapse. Figure S3: (A) Progression free survival for all DLBCL/HGBL patients stratified by baseline ctDNA concentration. (B) Progression free survival for treatment naive DLBCL/HGBL patients stratified by R‐IPI (no patients had ‘very good’ R‐IPI designation). Figure S4: (A) Sensitivity and detection limit for collapsed UMI‐family data (sized 3 or more family members) for 0.2% mix of control DNA from two individuals (101 SNP sites represented). (B) To assess background error rates, nucleotide sites 3’ and 5’ of the interrogated SNPs were analysed for the most frequent ‘alt base’ call (202 sites). In only one instance was an alternate base called, representing an allele frequency approximately 8‐fold lower than the deemed limit of detection (0.2%). Figure S5: Comparison of variants detected by whole exome sequencing (WES) of tissue biopsy versus deep sequencing of ctDNA illustrated by individual subjects. Light blue, variants detected on the 42‐gene panel with ctDNA input; Light green, variants detected by WES of tissue biopsy for the same 42 genes; Dark green, additional variants detected by WES of tissue biopsy but not represented in the 42‐gene ctDNA panel. Also represented are the LymphGen classifications based on ctDNA and WES data. GCB versus non‐GCB status is annotated according to Hans classifier. Figure S6:Histological features of patient #16 at diagnosis with DLBCL and relapse with Hodgkin lymphoma (x400). [file JHA2-6-e70126-s001.pdf]

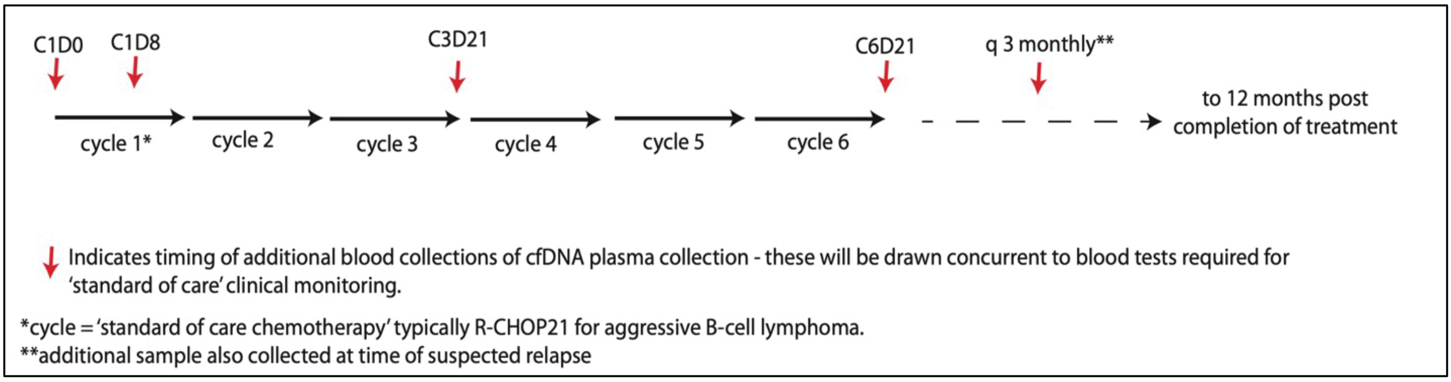

Supplementary Figure S1. Intended schedule for blood draws for plasma ctDNA analysis.

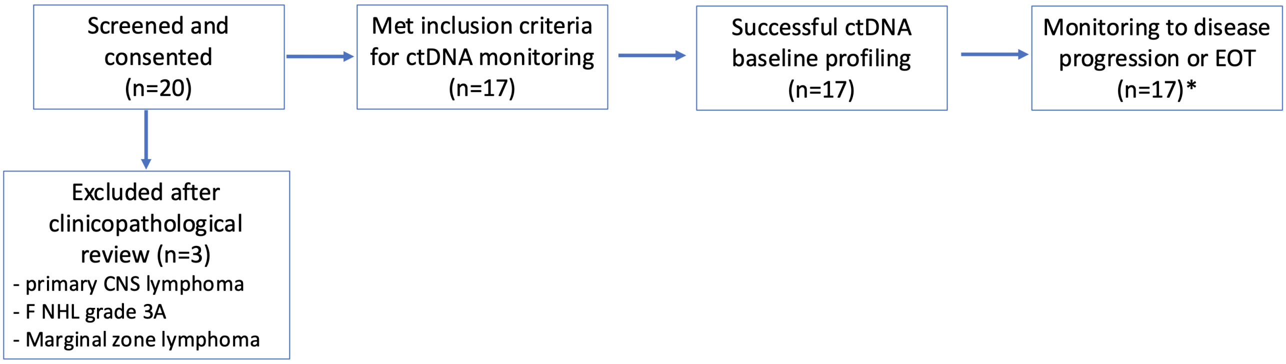

Supplementary Figure S2. Consort diagram of patients participating in the study. EOT, end of treatment. *includes patient who had baseline and EMR samples collected, missed EOT collection but continued to provide study samples at representation with Hodgkin relapse.

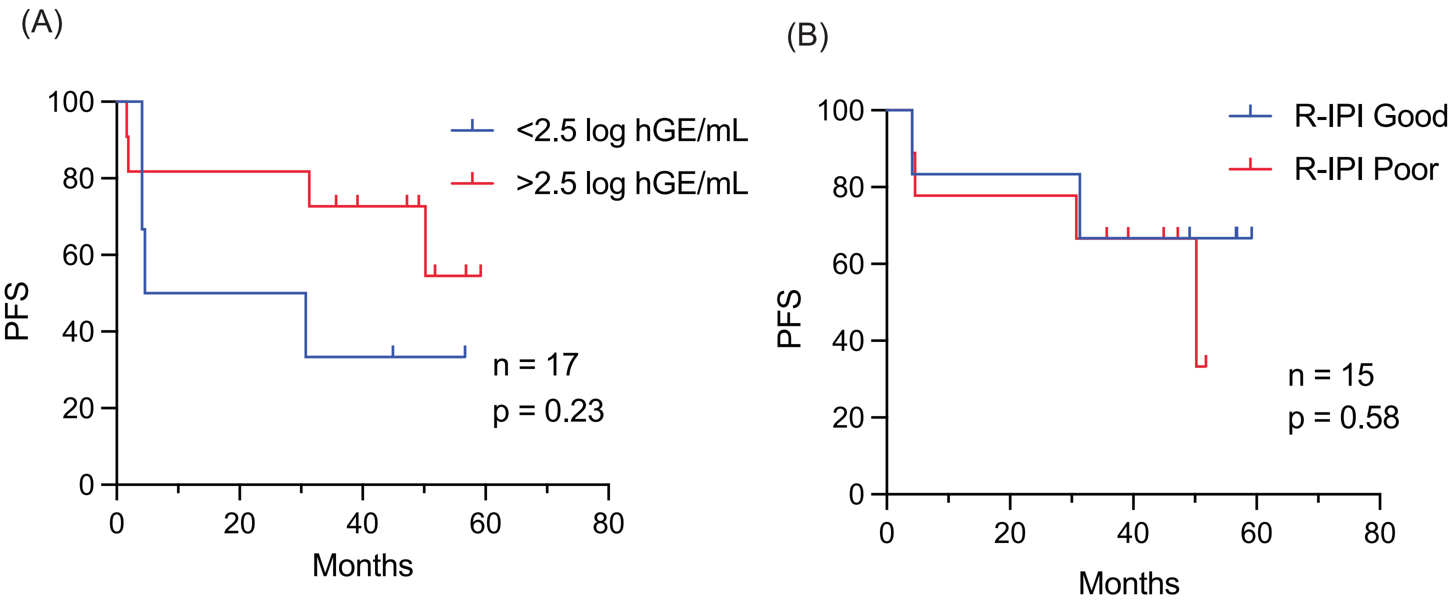

Supplementary Figure S3. (A) Progression free survival for all DLBCL/HGBL patients stratified by baseline ctDNA concentration. (B) Progression free survival for treatment naive DLBCL/HGBL patients stratified by R-IPI (no patients had 'very good' R-IPI designation).

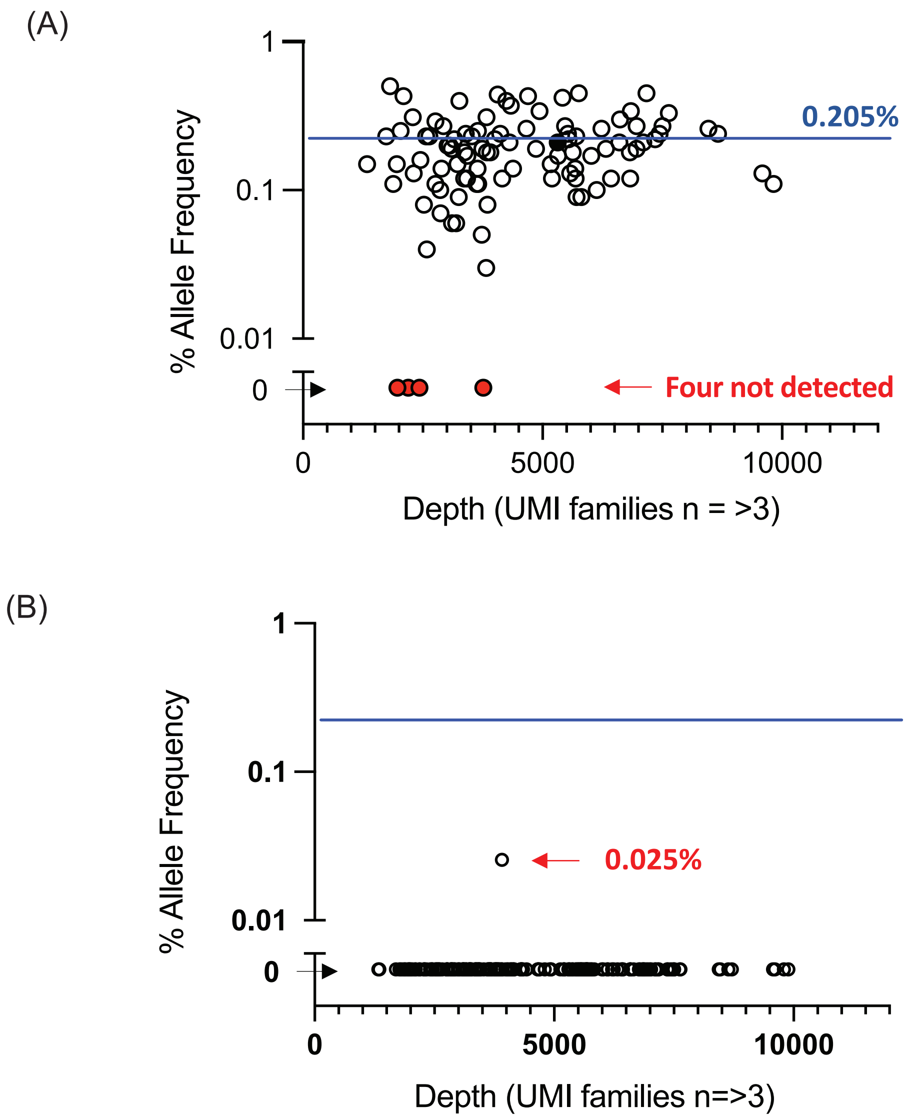

Supplementary Figure S4. (A) Sensitivity and detection limit for collapsed UMI-family data (sized 3 or more family members) for 0.2% mix of control DNA from two individuals (101 SNP sites represented). (B) To assess background error rates, nucleotide sites 3' and 5' of the interrogated SNPs were analysed for the most frequent 'alt base' call (202 sites). In only one instance was an alternate base called, representing an allele frequency approximately 8-fold lower than the deemed limit of detection (0.2%).

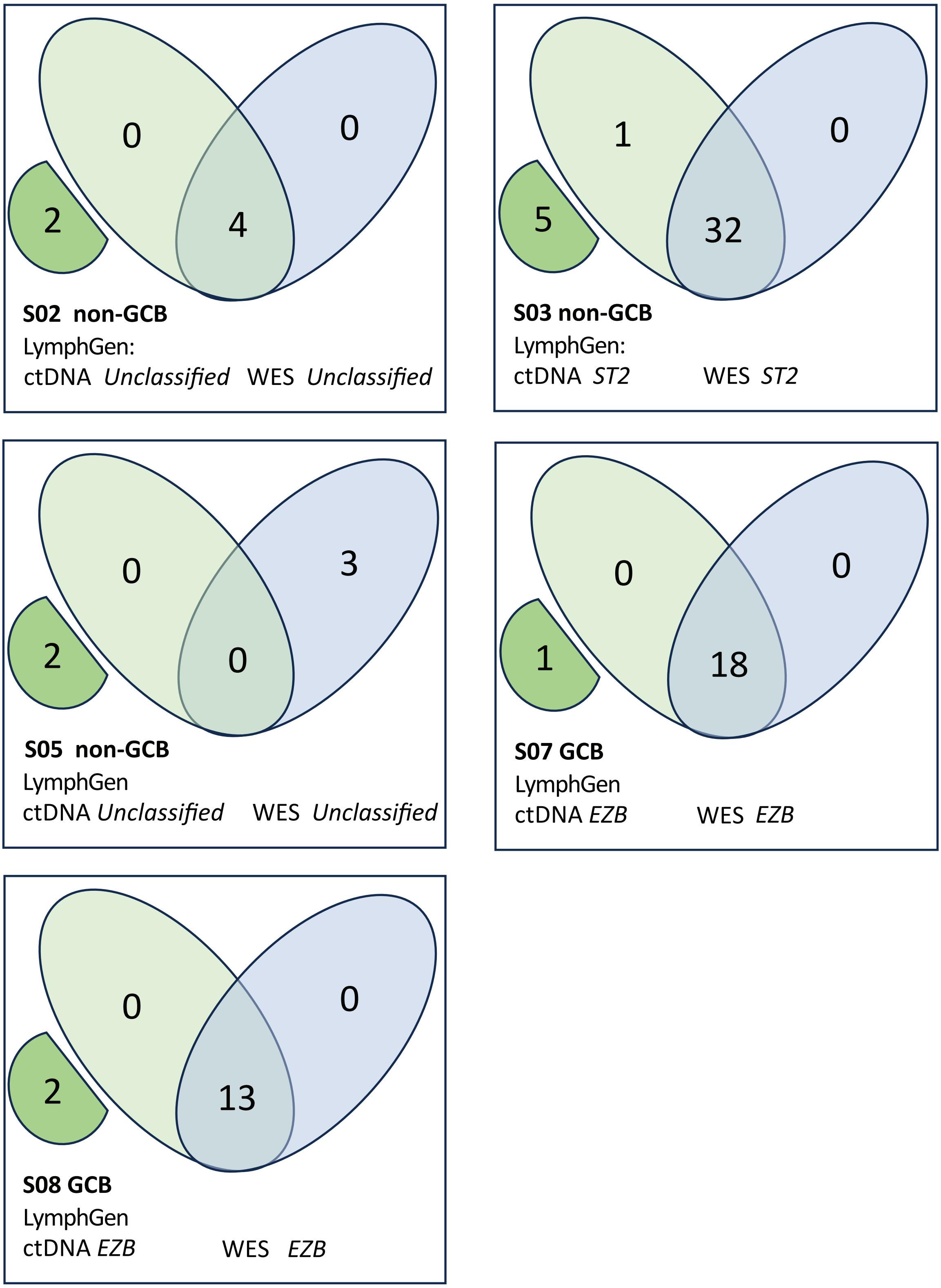

Supplementary Figure S5. Comparison of variants detected by whole exome sequencing (WES) of tissue biopsy versus deep sequencing of ctDNA illustrated by individual subjects. Light blue, variants detected on the 42-gene panel with ctDNA input; Light green, variants detected by WES of tissue biopsy for the same 42 genes; Dark green, additional variants detected by WES of tissue biopsy but not represented in the 42-gene ctDNA panel. Also represented are the LymphGen classifications based on ctDNA and WES data. GCB versus non-GCB status is annotated according to Hans classifier.

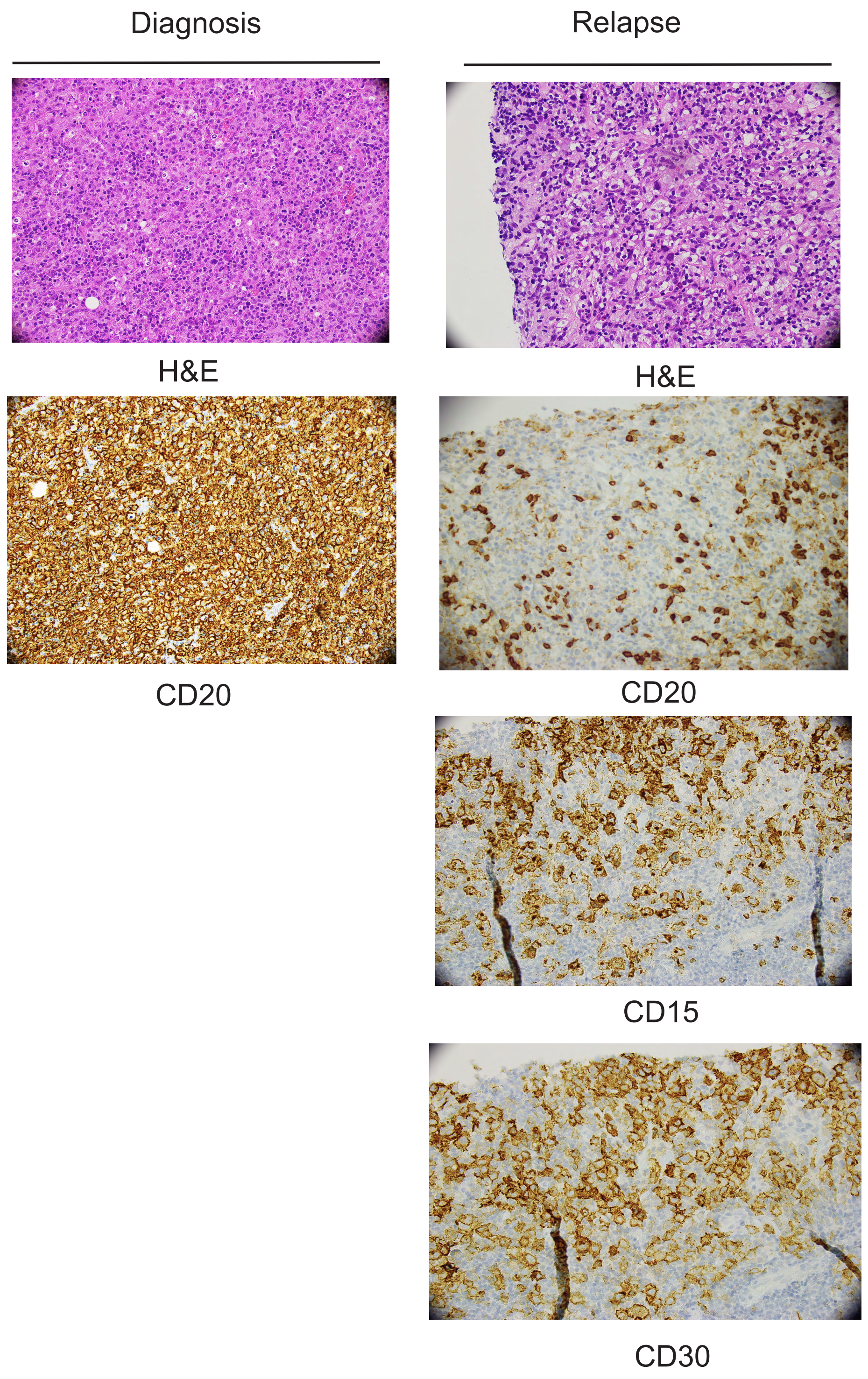

Supplementary Figure S6. Histological features of patient #16 at diagnosis with DLBCL and relapse with Hodgkin lymphoma (x400).
