# Supplementary material for: Dynamic Profiling of Cell Free Tumour DNA in Aggressive B‐Cell Lymphoma From Diagnosis to Transformation at Relapse
Source: EJHaem. 2025 Aug 19;6(4):e70126. doi: 10.1002/jha2.70126 (PMC12363405; doi:10.1002/jha2.70126)
Supplement: Supplementary file 2 — Table S1: Gene list (n=42) panel selected for assessment by ctDNA assay. An additional set of 1040 SNP genomic loci (distributed throughout the genome) was incorporated to facilitate CNV analysis. The inclusion of a SNP‐based CNV backbone also permitted the detection of mis‐labelled specimens using genotype information inherent to the sequence data to match samples as a quality control step. The total targeted region size for the design was 148.2 kbp, tiling density was ∼3x and total number of unique probes was 12,701. [file JHA2-6-e70126-s003.docx]

| ARID1A | CDKN2B | IGLL5 | PAX5 | TP53 |
| --- | --- | --- | --- | --- |
| B2M | CIITA | IRF4 | PIM1 | XPO1 |
| BCL2 | CREBBP | ITPKB | PRDM1 |  |
| BCL6 | EP300 | KMT2D | PRKCD |  |
| BRAF | EZH2 | BORCS8-MEF2B | RB1 |  |
| CARD11 | FAS | MFHAS1 | SOCS1 |  |
| CD58 | FBXO11 | MYC | STAT6 |  |
| CD79A | FOXO1 | MYD88 | TCF3 |  |
| CD79B | GNA13 | NOTCH1 | TNFAIP3 |  |
| CDKN2A | ID3 | NOTCH2 | TNFRSF14 |  |

**Supplementary Table S1.** Gene list (n=42) panel selected for assessment by ctDNA assay. An additional set of 1040 SNP genomic loci (distributed throughout the genome) was incorporated to facilitate CNV analysis. The inclusion of a SNP-based CNV backbone also permitted the detection of mis-labelled specimens using genotype information inherent to the sequence data to match samples as a quality control step. The total targeted region size for the design was 148.2 kbp, tiling density was ~3x and total number of unique probes was 12,701.
