# Supplementary material for: Dynamic Profiling of Cell Free Tumour DNA in Aggressive B‐Cell Lymphoma From Diagnosis to Transformation at Relapse
Source: EJHaem. 2025 Aug 19;6(4):e70126. doi: 10.1002/jha2.70126 (PMC12363405; doi:10.1002/jha2.70126)
Supplement: Supplementary file 3 — eJHaem_Waltham et al_Supplementary Methods_R1.docx [file JHA2-6-e70126-s002.docx]

**Supplementary Methods**

*Sample collection and processing*

Whole blood (10-20mL) was collected into Streck Cell-Free BCT^®^ tubes (La Vista, NE, USA) with plasma separated by centrifugation for stabilization (generally within 3-4 hours of collection). Buccal swabs were performed using ORACollect^®^ DNA collection kits (DNA Genotek Inc, ON, Canada) as per manufacturer’s instructions. Plasma and buccal DNA was purified using commercial isolation kits (Qiagen, Venlo, Netherlands) and quality metrics performed using Agilent TapeStation (Agilent Technologies, CA, USA). For analysis of tumor samples, DNA was extracted from formalin fixed paraffin embedded (FFPE) specimens and sequenced using Agilent Clinical Research Exome capture (CRE Ver 1 or 2).

*Next generation sequencing (NGS) of cell free tumor (ct)-DNA and analysis*

Input cell free (cf) DNA for NGS library preparation was 40 to 50 ng, harvested from (on average) 8 mL Streck^®^ plasma (i.e., two Streck Cell-Free BCT^®^ tubes [La Vista, NE, USA]). Libraries were manually built using Agilent XTHS reagents (Agilent Technologies, CA, USA) incorporating unique molecular identifiers (UMI; 10nt bp) to enable digital error correction as we have described previously^16,17^. Minimal PCR cycles were used at both pre- and post-hybridization steps in the protocol to maximise diversity of represented source DNA molecules. Illumina 2 x 150bp paired sequencing was used. In addition to specific targeted genes (main text and *Supplementary Table S1*), 1040 single nucleotide polymorphism (SNP) genomic regions broadly scattered over the genome were selected to function as a copy number variation (CNV) backbone and allow matching of specimens using genotype information as a quality control step^34^. The total targeted region size for the design was 148.2 kbp, tiling density was ~3x and number of unique probes was 12,701. NGS was performed on Illumina HiSeq or NovaSeq instruments at the Australian Genome Research Facility. Typically, 500-fold enrichment for the DLBCL-baited regions was observed across all plasma and buccal DNA samples; depth of sequencing in baited regions averaged 5000x once UMI families were de-multiplexed. In addition to *in silico*-based sensitivity estimation using *BAMSurgeon^35^*, mixing experiments utilizing DNA from two ethnically diverse donors utilized known SNP differences to test limits of detection. (Supplementary Figure S3).

*Bioinformatics and NGS data analysis*

An in-house bioinformatic pipeline was used as described previously^16,17^, incorporating tumor/normal (buccal gDNA) and including UMI de-duplexing and consensus calling using fgbio (Fulcrumgenomics /fgbio), bwa for alignment to human genome (hg19), VarDict^19^ for variant calling and CNVkit for copy number analysis^20^. Variant calls in samples were manually curated and confirmed by inspecting BAM files in Integrative Genomics Viewer (IGV)^21^. Phased somatic variants (paired within 100 nucleotide base pairs) were identified from BAM files and verified in IGV. NGS summary data and mutational plots were visualized using BoutrosLab.plotting.general (version 7.0.3) and commercial software (including GraphPad Prism, Ver 10 and MSPower BI, Ver 2.131). VarDict^19^ was used for variant calling and a minimum four Alt reads for the variant in question was required for demultiplexed BAM files, using UMI families with three or more members. Typically, 500-fold enrichment for the DLBCL-baited regions was observed across all plasma and buccal DNA samples; depth of sequencing in baited regions averaged 5000x once UMI families were de-multiplexed. Other VarDict metrics such as QUAL (>100), ODDRATIO (values 1 to <1.5) and PMEAN (>19.5) were used to filter out strand bias and potential end read errors from sequence data. Somatic mutations were readily distinguished from germline variants due to buccal sequencing and running VarDict in paired mode^19^. For every ctDNA timepoint, this standard analysis procedure used a uniform set of curation rules to identify variants with a VAF cut-off of >0.2%. This lower cut-off was rationalized from sensitivity and specificity experiments as presented in Supplementary Figure S3. Total ctDNA burden, expressed as hGE/mL plasma, was calculated from the measured ng/mL cfDNA concentrations and SNV variant allele frequencies^9^.

Phased somatic variants (for this study, paired within 100 nucleotide base pairs) were identified from BAM files (as generated for the standard analysis as described above) and verified in IGV. These were used to derive a more sensitive MRD detection procedure as first described by Kurtz et al. 2022^18^. For this study, the phased variants were exclusively identified from pre-treatment timepoint plasma sample. Sensitivity for this more sensitive measurable residual disease (MRD) detection was only restricted by sequencing depth, however in this case BAMs with UMI families of one or more family members were used (Fulcrumgenomics /fgbio). While depth of sequencing varied somewhat across capture sites, the phased variant approach usually afforded 40 to 100-fold more sensitivity than the standard ctDNA detection approach.

Variant annotation and pathogenicity were assigned using OpenCRAVAT^36^ which designates variant type along with an impact on protein structure (e.g., stop-gain, frame-shift deletions/ insertions, and complex substitutions) as well as integrating online data- base information, such as ClinVar (version 2022.06.14) and COSMIC (version 94.0.0). NGS summary data and mutational plots were visualized using BoutrosLab.plotting.general (version 7.0.3) and commercial software (including GraphPad Prism, Ver 10 and MSPower BI, Ver 2.131).

References

34. Wang, P.P., Parker, W.T., Branford, S. & Schreiber, A.W. BAM-matcher: a tool for rapid NGS sample matching. *Bioinformatics* **32**, 2699-2701 (2016).

35. Ewing, A.D.*, et al.* Combining tumor genome simulation with crowdsourcing to benchmark somatic single-nucleotide-variant detection. *Nat Methods* **12**, 623-630 (2015).

36. Pagel, K.A.*, et al.* Integrated Informatics Analysis of Cancer-Related Variants. *JCO Clin Cancer Inform* **4**, 310-317 (2020).
